# Supplementary material for: Challenges and Lessons Learned from a Field Trial on the Understanding of the Porcine Respiratory Disease Complex
Source: Vaccines (Basel). 2025 Jul 9;13(7):740. doi: 10.3390/vaccines13070740 (PMC12299284; doi:10.3390/vaccines13070740)
Supplement: Supplementary file 1 [file vaccines-13-00740-s001.zip › SupFig_S2_Linear regression between ISU-VDL PCR data and NanoString reads for specific pathogens.pdf]

Sup. Fig S2

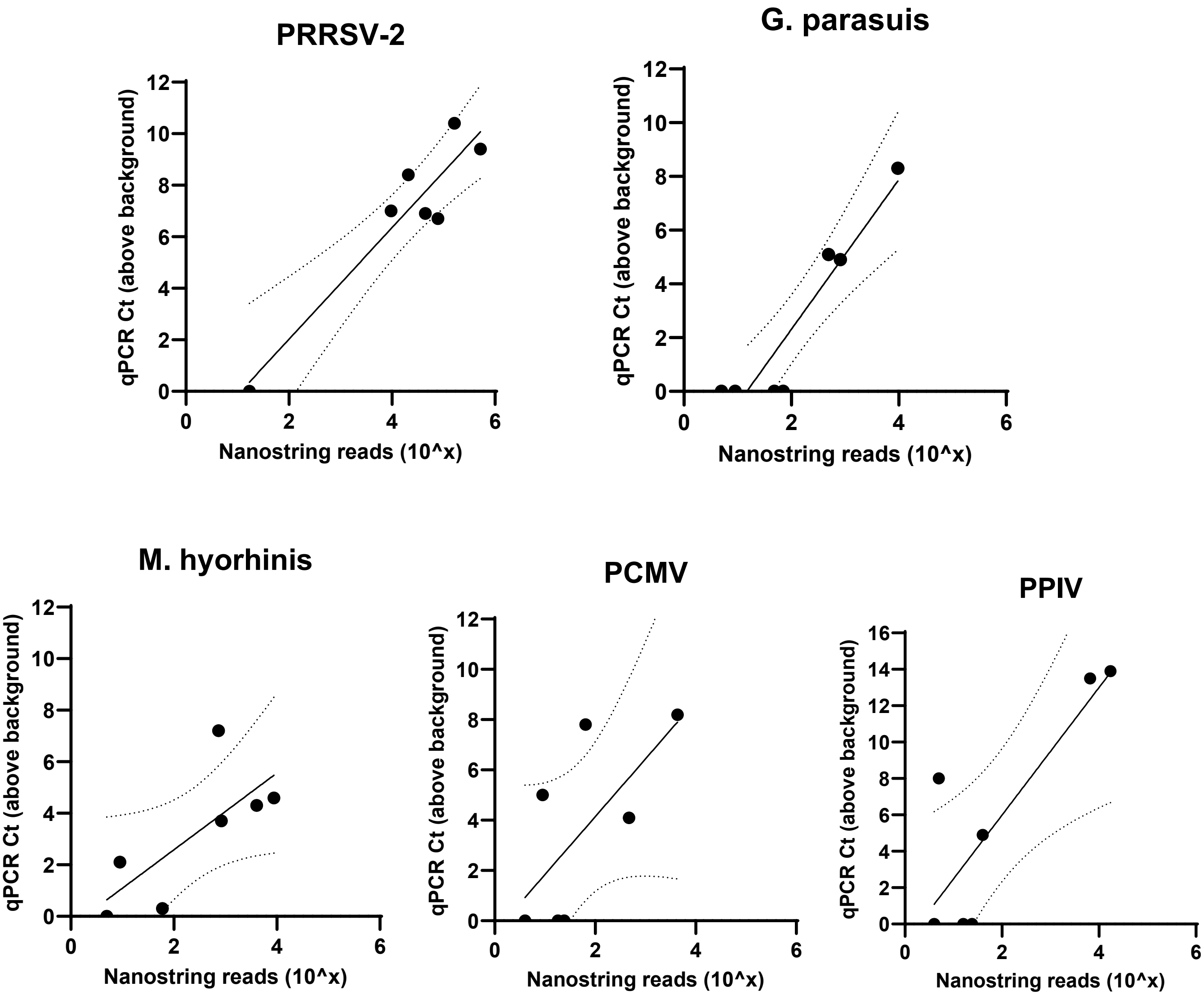

Correlation (one-tailed Spearman)

PRRSV-2: p=0.0694

G. parasuis: p=0.0143

M. hyorhinis: p=0.0331

PCVM: p=0.0762

PPIV: p=0.0524

Linear regression

PRRSV2: p= 0.001, R^2: 0.88

G. parasuis: p= 0.01, R2: 0.88

M. hyorhinis: p= 0.057, R^2: 0.55

PCMV: p= 0.101, R^2: 0.45

PPIV: p= 0.01, R^2: 0.70
