# Supplementary material for: Challenges and Lessons Learned from a Field Trial on the Understanding of the Porcine Respiratory Disease Complex
Source: Vaccines (Basel). 2025 Jul 9;13(7):740. doi: 10.3390/vaccines13070740 (PMC12299284; doi:10.3390/vaccines13070740)
Supplement: Supplementary file 1 [file vaccines-13-00740-s001.zip › Table S4_ Virus shedding in nasal swabs.pdf]

Table S1. NanoString custom codeset of 26 genes for pathogen detection

Gene	Accession No.	Class Name
PRRSV_M	KX192112.1	Endogenous
PRRSV VR-2332 ORF1ab	NC_038291.1	Endogenous
Porcine respiratory coronavirus PRCV_ORF1a	KR270796.1	Endogenous
Swine IAV HA H1	AB762402.1	Endogenous
Swine IAV_HA_H3	KC471441.1	Endogenous
Swine IAV_NA_N1	AM920729.1	Endogenous
Swine IAV_NA_N2	AF225538.1	Endogenous
PCV-2_ORF1	NC_005148.1	Endogenous
PCV-3 caspid	BMR83_gp1.1	Endogenous
SuHV-1_UL30	DUL34gfp_UL30.1	Endogenous
Porcine Rubulavirus (PoRV)_L	NC_009640.1	Endogenous
Nipah_L	NC_002728.1	Endogenous
PCMV U38	P379_gp32.1	Endogenous
PPV_caspid	NC_001718.1	Endogenous
TTSuV_ORF1	JX173482.1	Endogenous
Mycoplasma hyopneumoniae rpoC	MHJ_RS03275.1	Endogenous
Glaesserella parasuis rpoD	HAPS_RS05805.1	Endogenous
Streptococcus suis rpoD	SSUBM407_1331.1	Endogenous
Bordetella bronchiseptica rpoD	BN112_0963.1	Endogenous
Actinobacillus suis rpoD	ASU1_RS10885.1	Endogenous
Actinobacillus pleuropneumoniae rpoD	APL_RS07780.1	Endogenous
Pasteurella multocida rpoD	DR93_RS03555.1	Endogenous
Trueperella pyogenes rpoC	CQ11_RS04735.1	Endogenous
Mycoplasma hyorhinis rpoD	MOS_RS02265.1	Endogenous
Porcine parainfluenza virus HN	KT749884.1	Endogenous
Sus_scrofa_ABCF1	NM_001123069.1	Housekeeping

Table S4. Virus shedding in nasal swabs

PIG #	Day 28 Ct	Day 49 Ct	PIG #	Day 28 Ct	Day 49 Ct
**1**	≥37	≥37	**31**	35.0	≥37
**2**	≥37	≥37	**32**	≥37	≥37
**3**	≥37	≥37	**33**	35.3	≥37
**4**	≥37	≥37	**34**	36.4	≥37
**5**	≥37	≥37	**35**	≥37	≥37
**6**	≥37	≥37	**36**	≥37	≥37
**7**	≥37	≥37	**37**	≥37	≥37
**8**	≥37	34.0	**38**	35.7	≥37
**9**	≥37	≥37	**39**	≥37	≥37
**10**	≥37	≥37	**40**	≥37	≥37
**11**	≥37	≥37	**41**	33.8	≥37
**12**	≥37	≥37	**42**	≥37	≥37
**13**	≥37	≥37	**43**	34.9	≥37
**14**	≥37	≥37	**44**	≥37	≥37
**15**	≥37	≥37	**45**	35.9	≥37
**16**	≥37	≥37	**46**	≥37	≥37
**17**	≥37	≥37	**47**	≥37	≥37
**18**	≥37	≥37	**48**	≥37	≥37
**19**	≥37	≥37	**49**	≥37	≥37
**20**	≥37	≥37	**50**	≥37	≥37
**21**	≥37	≥37	**51**	≥37	≥37
**22**	≥37	≥37	**52**	≥37	≥37
**23**	≥37	≥37	**53**	≥37	≥37
**24**	≥37	≥37	**54**	≥37	≥37
**25**	≥37	≥37	**55**	34.8	≥37
**26**	≥37	≥37	**56**	≥37	≥37
**27**	≥37	36.9	**57**	≥37	≥37
**28**	≥37	≥37	**58**	≥37	≥37
**29**	33.8	≥37	**59**	≥37	≥37
			**60**	≥37	31.8
