# Supplementary figures and images for: Challenges and Lessons Learned from a Field Trial on the Understanding of the Porcine Respiratory Disease Complex
Source: Vaccines (Basel). 2025 Jul 9;13(7):740. doi: 10.3390/vaccines13070740 (PMC12299284; doi:10.3390/vaccines13070740)

**A**

# Sup Fig S1

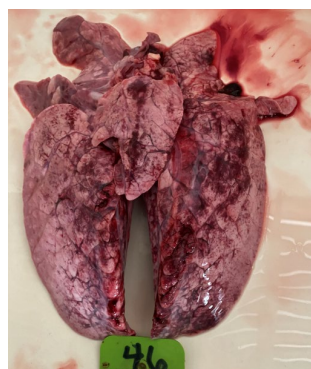

52.5%

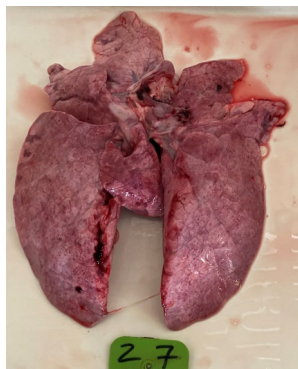

75%

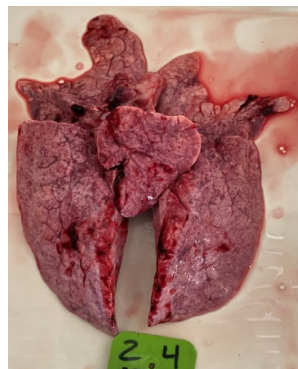

87%

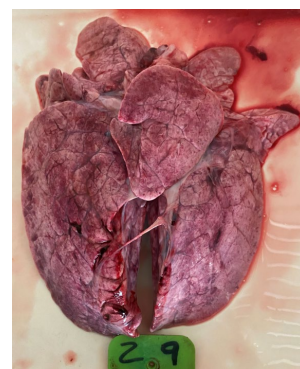

100%

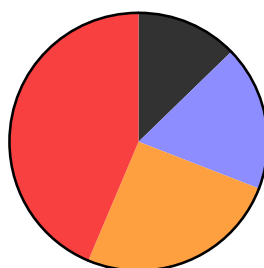

12.7% <79%  
18.1% 80-89%  
25.4% 90-99%  
43.6% 100%

**B**

Total=55

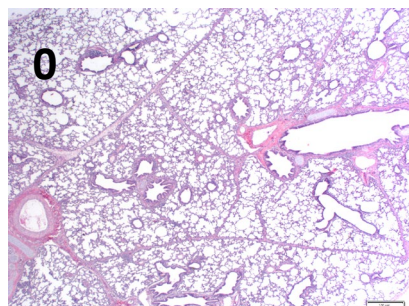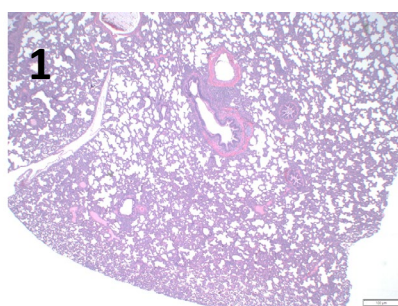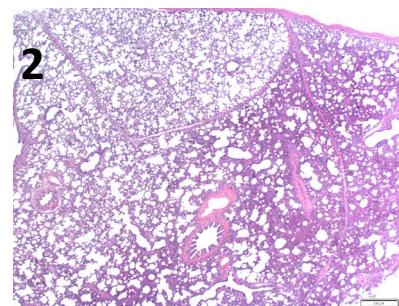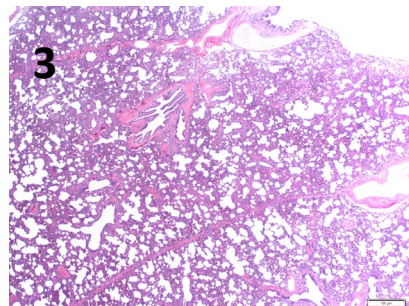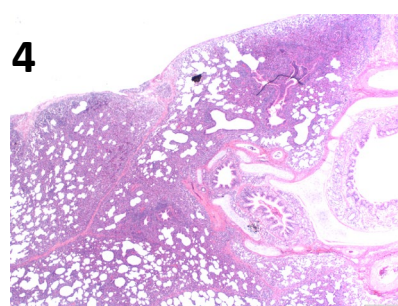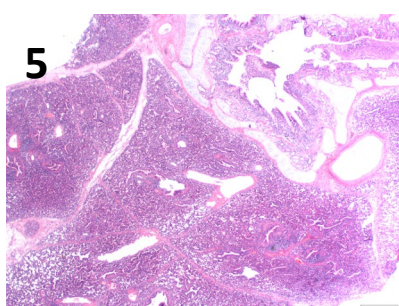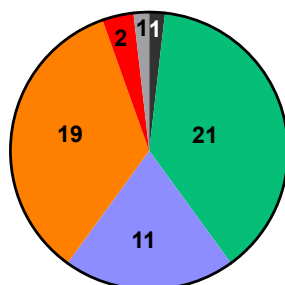

1.8% Score 0  
38.1% Score 1  
20.0% Score 2  
34.5% Score 3  
3.6% Score 4  
1.8% Score 5

Total=55

Supplement: Supplementary file 1 [file vaccines-13-00740-s001.zip › SupFig_S1_Lung pathology.pdf]
